# Life threatening self starvation; a case report

**DOI:** 10.1186/1756-0500-6-36

**Published:** 2013-01-31

**Authors:** Chaturaka Rodrigo, Thushani Henegama, Raveen Hanwella

**Affiliations:** 1 Department of Clinical Medicine, Faculty of Medicine, University of Colombo, Colombo, Sri Lanka; 2 Department of Psychological Medicine, Faculty of Medicine, University of Colombo, Colombo, Sri Lanka

## Abstract

**Background:**

Obsessive compulsive disorders are a complex group that can have a variety of manifestations. Many authors now describe an obsessive compulsive spectrum disorder where many other specific diagnostic entities such as trichotillomania, tic disorders and body dysmorphic disorder are considered to be related and linked disorders.

**Case presentation:**

We report a case of a twenty two year old Sri Lankan male who presented with life threatening self starvation due to severe obsessive compulsive disorder. The diagnosis was not considered till late due to the atypical presentation of the patient. While his symptoms bordered on a delusional psychosis, a decision was made to treat him as for obsessive compulsive disorder with behavioural therapy which was successful in the end.

**Conclusions:**

In analysis of a patient with severe anorexia, the psychological causes should not be forgotten. In fact, if the feeding pattern of the patient was observed at the beginning, unnecessary investigating and life threatening worsening of the condition could have been avoided.

## Background

Research in to obsessive compulsive disorder (OCD) over the last 15 years have raised the possibility that it co-exists with many other disorders considered to have a separate organic and/or a psychological basis [1]. These include; delusional disorders, eating disorders, impulse control disorders, pica, pathological gambling and Tourette syndrome. This led to a hypothesis of obsessive compulsive spectrum disorder (OCSD) [1-6]. The classic description of OCD as defined in International Classification of Diseases −10 (ICD-10) involves the presence of obsessions (recurrent, intrusive, unwanted thoughts, images or impulses) and compulsions (repetitive behaviors either covert or overt). However, in OCSD, such behaviors may co exist and sometimes contribute to the pathogenesis of the other disorders mentioned above. Furthermore, the characteristic description of OCD states that the obsessions and compulsions are an agony to the patients and that he or she is aware that it is a redundant behaviour. However, considering OCSD, some of the obsessions are so ingrained to the patients’ beliefs that they become ‘delusional’ in nature. This can lead to serious consequences that can be life threatening in some patients. We present a case of a twenty two year Sri Lankan male who presented with severe OCD associated with delusional thoughts that responded well to behaviour therapy rather than antipsychotics.

## Case presentation

A twenty two year old Sri Lankan male presented to the outpatients department of the National Hospital of Sri Lanka complaining of shortness of breath. Noting that the patient was severely emaciated, he was requested to get admitted to a medical ward for further investigations. While in ward his main complaint was recurrent shortness of breath and a bloated feeling that was brought upon by ingestion of food. He also complained of severe constipation and maintained that he had cut down on eating due to this. He did not have symptoms such as pyrexia, symptoms of thyrotoxicosis, bleeding per rectum, chronic diarrhoea and lumps on palpation anywhere in the body. He had no medical history suggestive of chronic infections such as tuberculosis, metabolic disorders such as diabetes, immunodeficiency disorders and primary gastrointestinal disorders such as celiac disease or chronic pancreatitis. There were no inherited organic illnesses in the family, risk behaviors for Human Immunodeficiency Virus (HIV) infection or a history of substance abuse. He was extremely wasted with a body mass index (BMI) of 11.1 kg/m^2^ (weight 27 kg, height 1.56 m). He did not have any overt signs of micronutrient and vitamin deficiencies. The cardiovascular examination was normal and the blood pressure was 110/70mmHg. The respiratory, abdomen and nervous system examinations did not reveal any clinically significant findings.

He was investigated extensively to identify an organic pathology to explain the emaciation. The haemoglobin was 11.5 g/dl with normal white blood cell and platelet counts. The blood picture was also unremarkable apart from features of iron deficiency. The erythrocyte sedimentation rate was 3mm in first hour and the liver function tests and renal function tests were within normal limits. The fasting blood glucose was 3.3 mmol/l and the serum amylase was 327 U/l. The total serum protein level was 68 g/l with an albumin level of 48 g/l suggesting a more recent cause for weight loss as the protein content was not grossly reduced. The electrolyte profile was normal apart from a hyponatremia (128 meq/l) and there were no significant findings on the chest roentgenogram or the ultrasound scan of abdomen. An upper gastrointestinal endoscopy was arranged and revealed a normal oesophagal, gastric, duodenal and upper jejunal mucosa. Multiple biopsies taken at different sites were within normal histological limits. The HIV screen was negative and the thyroid hormone profile was also normal.

At this point it was noted that the patient was avoiding food. He constantly complained of food causing uncomfortable abdominal distention, epigastric pain and shortness of breath. He was then referred to the University Psychiatry Unit of the National Hospital of Sri Lanka for further assessment for an underlying psychiatric morbidity.

On the initial psychiatric interview it became clear that these recent constellation of symptoms had started three months back after he became constipated following treatment for an infected eczematous rash. He was heavily inconvenienced by the abdominal distension plus the difficulty in passing stools and therefore tried to avoid food to minimize the discomfort. Later, he became preoccupied with the idea of keeping his body free of symptoms by avoiding food. He was having recurrent intrusive fears of ingested food being retained in the body without him being able to defecate due to constipation. This led to secretive disposal of food and resultant weight loss. However, he was not convinced that his beliefs were irrational. His idea of food being retained in the body and accumulating causing discomfort due to the potential inability to defecate, bordered on a delusional thought. Apart from being withdrawn and wasted with a blunt mood, the rest of the mental state examination was normal. Family members have noted that he was withdrawn and smiling to self on occasions over the last 4–5 years. He was unemployed, spent most of his time at home but didn’t help out with the daily family chores. He did not have any special interests, hobbies or religious interests. The differential diagnosis at this point included; OCSD with a delusional component, a prodrome of schizophrenia or simple schizophrenia.

A probable diagnosis of a type of OCSD was supported by a history of symptoms suggestive of OCD . For the past two years he had repetitive behaviours such as hand washing due to obsessional thoughts of uncleanliness. Rituals related to hand washing and eating led to significant obsessive slowness sometimes exceeding two hours to complete a meal. In addition to excessive washing, he used to stare at the tap prior to washing as a part of the ritual, sometimes up to an hour. Considering the overall picture it was decided to treat him with cognitive behavioural therapy (CBT) as for OCD instead of starting antipsychotic medication. Because of his poor physical health in ward treatment was necessary. Due to his food refusal he had to be sedated and fed via a nasogastric tube in the first few days. To avoid refeeding syndrome his biochemical parameters including electrolytes were regularly checked. Wernicke’s encephalopathy was anticipated and treated with thiamine. As he gradually gained his strength CBT was started early. The behavioural therapy consisted of exposure and response prevention. The exposure consisted of graded refeeding while being encouraged to face the anxiety associated with the obsessive fear of being unable to tolerate meals and constipation. After the initial inpatient stay of two weeks, therapy was carried out at home with the mother being the co-therapist. He was not started on any antipsychotic or anti depressant medications and was managed on CBT alone. At three weeks, his weight increased by 1 kg and after 3 months by 5 kg (BMI 13.1 kg/m^2^). The average time taken for a meal reduced from 90 minutes to 20 minutes. With less preoccupation about his meals/bowels habits, his social interactions and involvement with other members of the family improved markedly. At six months follow up his improvement continued with further weight gain (BMI 15.8 kg/m^2^). His interactions with family members and neighbours had also improved. He remained unemployed but was fulfilling his daily household chores and was considering active employment. The excellent response to behavioural therapy within a relatively short time span was surprising but was a vindication of the diagnosis of OCD.

## Discussion

Recent onset rapidly worsening emaciation of a previously ‘healthy’ young adult male raise the possibility of a sinister underlying organic pathology such as a hormonal disorder, haematological (lymphoma/leukaemia) or solid organ malignancy (e.g. gonadal tumour), infectious diseases such as tuberculosis or HIV, severe substance abuse with food neglect or a primary gastrointestinal disorder such as celiac disease or malabsorption due to pancreatic disease (autoimmune pancreatitis) [7]. However many of these diagnoses could have been excluded by simple observation of his food habits. Many primary gastrointestinal disorders that cause a significant weight loss do so because of chronic diarrhoea. This patient was persistently complaining of constipation. Other disorders with a predominantly psychiatric pathogenesis such as anorexia nervosa or laxative abuse are not common among males though it should have been considered in the initial differential diagnosis. The patient was extensively investigated including an upper GI endoscopy that excluded many likely organic illnesses. OCD have been of interest to many researchers over the last decade. Two major debates are focused on OCD at the moment a) whether it should be classified as an anxiety disorder and b) the existence of obsessional compulsive spectrum disorders (OCSD). The discussion of this case report is more relevant to the latter.

Many authors have pointed out the similarities in symptomatology of OCD and diagnoses such as trichotillomania, Gilles de la Tourette syndrome, body dysmorphic disorder and pica [5,6,8,9]. All these disorders are characterized by undesirable body focused behaviors that are repetitive in nature. Regarding pathophysiology, in a review of evidence, Stein shows a number of commonalities between the classic OCD and putative OCSDs varying from genetics (tic disorders and OCD), serotonin and dopamine neurotransmitter systems and autoimmunity (OCD and trichotillomania, tic disorders) [3]. In addition, these disorders and OCD share similarities in response to treatment as well [10,11]. In one of the largest studies conducted to-date, Stein and Lochner [12] assessed 417 patients with a diagnosis of classic OCD, for co-existence of other OCSDs. They also compared the results with patient populations with panic disorder and social anxiety disorder (based on the above mentioned hypothesis that OCDs share common features with anxiety disorders also). It was observed that certain OCSDs like trichotillomania and self-injury were commoner in the classic OCD group while certain other conditions such as hypochondriasis and body dysmorphic disorder were commonly observed in the anxiety disorder groups. These facts show that the entity of OCD as defined previously may be part of a wider spectrum. Whether this spectrum also merges in to the anxiety disorder group is still doubtful.

The situation in our patient was unusual in that it did not fit in to the characteristic OCD pattern. The thought of food being retained in the body leading continuous bloating and the inability to defecate was bordering on a delusion or an overvalued idea. The patient was not fully convinced that his belief was senseless. In fact, it led to a life threatening self starvation with a rapid weight loss over a period of three months. Delusional beliefs in OCD have been reported before and some authors believe as the disease becomes severe, the patient loses the conviction that his obsessional thoughts are senseless [13]. This leads to severe disability. As a proportion it is estimated that 5% of OCD patients lose insight regarding the obsessions. This group is identified as a separate subgroup in DSM-IV TR [14].

During the past two decades cognitive behaviour therapy (CBT) and pharmacotherapy that inhibit the serotonin reuptake, have emerged as being effective in OCD. Two groups of drugs, the tricyclic antidepressants (clomipramine) and the specific serotonin reuptake inhibitors (SSRI) have been shown to be effective in randomised trials. A combination of CBT and serotonin reuptake inhibitors gives the best results in clinical settings [15]. However, our problem with this patient was unique in that he refused oral intake of food let alone medication. The first priority was gradual refeeding with a nasogastric tube and as the malnutrition was corrected CBT was started. Both the patient as well as the parents preferred CBT to medication (probably influenced by the stigma attached to being on ‘psychiatric drugs’). Since he responded very well to CBT there was no need to start on drugs. Our experience highlights the importance of individualization of therapy to suit the needs and preferences of the patient.

## Conclusion

Of the many OCSDs so far described, we believe that our patient falls in to a category of OCD associated delusional disorder. It clearly distinguishes itself from a body dysmorphic disorder given the content of the delusional ideation. This case report highlights the importance of coming to a correct diagnosis after carefully weighing the pros and cons for a particular differential diagnosis. Given the historical factors and changes in pre-morbid personality, he could have been easily misdiagnosed and treated as a patient with prodromal or simple schizophrenia. It also highlights a rare life threatening form of OCD that is best described as a part of an OCSD as it does not fall within the classic description of OCD. Finally to the general physician, it highlights the importance of simple measures like observation of food habits in a patient presenting with weight loss and malnutrition. It would save a lot of time and effort in narrowing down the differential diagnosis.

## Consent

Written informed consent was obtained from the patient and his legal guardian (mother) for publication of this case report. Copies of the consent are available for review by the Editor-in-Chief of this journal.

## Competing interests

The authors declare that they have no competing interests.

## Authors’ contributions

CR wrote the first draft and did the literature survey. RH and TH were responsible for the clinical care of the patient. All authors contributed to and approved the final manuscript.

## Authors’ information

CR (MBBS) is lecturer in Medicine in the Department of Clinical Medicine, Faculty of Medicine, University of Colombo. TH (MBBS, MD) is senior registrar in psychiatry, in the University Psychiatry Unit of the National Hospital of Sri Lanka. RH (MBBS, MD, MRCPsyph) is consultant psychiatrist attached to the Department of Psychological Medicine, Faculty of Medicine, University of Colombo.
